# Report of Committee on Intelligence and Education1Read before the meeting of the Pennsylvania State Veterinary Medical Association, March 5, 1901.

**Published:** 1901-08

**Authors:** H. B. Felton

**Affiliations:** Philadelphia, Pa.


### REPORT OF COMMITTEE ON INTELLIGENCE AND EDUCATION.[1]

By H. B. Felton, V.M.D.,
PHILADELPHIA, PA.

The century that has just closed has almost spanned the history of veterinary education in Europe, and the last half of it has witnessed the inception and marvellous growth of this branch of medicine in the United States. All honor to the pioneers who blazed the way, who cleared the ground, and sowed the seed that at the dawn of the twentieth century has sprung up into a mighty army of trained and educated veterinarians, in whose hands are placed interests that not only represent untold millions of money, but which also tend to conserve animal life and human life! The last decade has been marked by a gradual widening of the field of the veterinarian. We have gained an eminence from which we have been able to command that wider outlook of which Bishop Potter has spoken so recently. How important, then, that we should recognize the necessity of broader tendencies in veterinary instruction, a subject which has been so ably treated by Dr. Pearson in his last address before the American Veterinary Medical Association. If the veterinarian is to be an animal engineer, as Dr. Pearson suggests—and we think the designation an excellent one—he must understand the animal machine thoroughly in health and in disease. He must know how to develop the highest possible efficiency of this mechanism either for the various forms of labor required of the domestic animals or for the production of milk or meat. In short, he must be a many-sided man, who has laid the foundations of his education broad and deep. It is just possible that in this broader field we shall find the salvation and the perplexity of our profession. We have arrived at an era when economical conditions are subject to sudden and startling changes. We have witnessed recently revolutions in many great and important industries. It is within the bounds of possibility that changes may come quickly which will involve the withdrawal of large numbers of horses from the industrial field and the substitution of machines which need no veterinarian to diagnose their ills. In the broader field of usefulness that lies before us, however, we see no need of decimating the ranks of our profession, and can per-

[1] Read before the meeting of the Pennsylvania State Veterinary Medical Association, March 5, 1901.

ceive a sphere where all may be employed if we will measure up to its requirements.

Among the institutions of learning that have recognized the necessity for the broader education of the veterinarian, the University of Pennsylvania has stood in the forefront. The veterinary profession owes a lasting debt of gratitude to Dr. Huidekoper, through whose efforts the Veterinary Department of the University was organized on such broad and liberal lines, and who, if he had been allowed to carry out certain plans, would have broadened its field of usefulness still more fully. This school is fortunate in having a devoted corps of able teachers, who have given so largely of their time for such inadequate pay, and in having a provost who appreciates thoroughly the value and importance of our profession, and is doing all that lies in his power to secure an adequate endowment, so that the school shall rest upon a firm financial basis, with ample money to compensate its instructors and to develop more fully certain departments in order to keep it fully abreast of the times. In the recent changes made in the University grounds the veterinary hospital has become somewhat sequestered—a fact which has considerably impaired its usefulness—and one of the most pressing demands at the present time is a change to a more desirable and more public location. It has been rumored that another veterinary school is about to be organized in Philadelphia in connection with one of our medical colleges. We earnestly hope that this will not be consummated. No city has ever been found yet large enough to support two successful veterinary schools. The experiment has been tried in Paris, in Edinburgh, in New York, Chicago, and Washington, and has been a failure in every case. It is very much the same thing as we see sometimes in small towns where there is a strong, flourishing church, and from some cause or other another church of the same denomination will be built, drawing a certain number of members from the one first established; and, as a consequence, where there was formerly one strong, vigorous church there will be two weak churches, neither of which can be adequately supported. We hope the members of this Association will bring all their influence to bear to discountenance this project. Let us rather do all in our power to strengthen the hands of what is to many of us our alma mater, and help to keep her in the place which rightfully belongs to her, as the leader of veterinary education in America. It is a matter of regret to many that Harvard University has decided to close her veterinary department after the present classes shall have been graduated.

We would regard this as a backward step had we not been informed that steps are being taken to have the school reorganized upon a broader and more enduring basis. We hope this may be effected, for the Veterinary School at the present day is a necessary adjunct to the University, and with this arm lopped off its field of usefulness must be necessarily restricted. As this is a time of retrospect and comparison of the present with the past, we thought it might be of interest to compare the condition of the farmer in the new century with what it was fifty years ago.

We quote from the *Philadelphia Record:* "The lessening of labor on the farms of this country cannot be appreciated except by those who have had experience on farms thirty or forty years ago; that is by a comparison of the present with the past. Half a century ago many of the breeds of horses, cattle, sheep, and swine were unknown. The fast trotter had not come into existence, while the running horses were from ten to twenty seconds slower in going a mile on the race-courses of that day if compared with the present records of time. There were no Jersey, Holstein, Galloway, Angus, or Guernsey breeds of cattle in this country, and the Shorthorn was known as the Durham. The Cotswolds held the lead among sheep, the Oxfords, Shropshires, and Hampshires (all down breeds) coming later. The Berkshire hog had been introduced, but it was not like the Berkshire of to-day. The Chester White, Yorkshire, Essex, and Suffolk hogs were years in winning public favor, while the Poland China is of much later date. Among poultry the Shanghai and Cochin were introduced about 1853, and from them have come by crossing many of our best breeds of poultry, such as Brahmas, Plymouth Rocks, Wyandottes, and different varieties of Cochins. The Embden and Toulouse geese, Pekin ducks, and some of the breeds of turkeys were unknown. The Percheron, Clydesdale, and Hackney horses were not improved to their present form even in Europe, and the horses used were lighter, but had more speed and endurance in hauling loads to market, as the farmer had to use the turnpike and country roads to forward his produce, even the roads being as primitive as the implements and wagons used. The animals of the present day give almost double the service derived from their kind half a century ago. Comparing the new year and the new century with 1850, the labor-saving implements that have been invented for service on the farm, the farmer of the present can ride when he plows, harrows, cultivates, plants, or harvests his crops. He can also perform more work in some lines of farming in a day than could formerly have been done in a week, and also at less cost. His produce can be sent to market in bulk, and in a few hours instead of days, and his wagons are not only stronger and lighter, but much cheaper. Wire has banished the old style worm-rail fence, and new varieties of grains, fruits, and vegetables have been introduced. In 1850 the strawberry was but little larger than a pea; the tomato was about the size of a walnut; the crab apple was in the lead; the Concord grape was unknown, and the peach and pear had not been greatly improved. Fruit-growing as a business had not made any progress, and thousands of bushels of apples rotted in orchards every year. In some sections the cattle, hogs, and sheep were expected to secure a large share of their food on unproductive fields, and the mast of the forests was considered a necessary adjunct in the feeding of hogs. Flocks of sheep were turned out to roam at will, being salted occasionally in order to have them come up once in a while to be counted. The cow bell was one of the equipments, as the animals would frequently stray away and were brought up at night to be milked. Now, the cow bell is not required, and the hog that is active enough to seek mast in the forests is not the kind wanted. Co-operation is also gradually entering into farming, though perhaps not noticed by many. One grain separator and cleaner drawn from farm to farm by a traction engine does the work for the entire neighborhood. The creamery and the condensed milk factory create markets at the doors of the farms. The farmer can buy butter cheaper from the creamery than he can make it from a small herd, and he combines with his neighbors in the purchase of pure-bred stock. Within the past year the corn-fodder shredder has been on its mission performing work for several farms, while fruit growers have organizations which give them advantages in shipping and selling. Before a decade passes away the larger share of the work on farms will be performed with electricity as the source of power. Already the wire fences serve the farmers to convey telephone messages from one farm to another, and free delivery of mails at the door will soon cease to be a novelty. The condition of the farmer has greatly changed in many respects. Railroads bring the farmer within an hour or more of the city, while fifty years ago at least two days were required to make such a journey. Free schools are in every township, and the roads are being made better every year. The farmer makes more actual profit than formerly, as he gets better prices for his produce compared with the cost, while the goods he purchases are much cheaper, many of the articles used at present being enjoyed only by a few as luxuries half a century ago.''

The question might be asked, How will the veterinarian be benefited by his relation to the farmer of the twentieth century? The answer may be given in the words of a certain writer: "The twentieth century farmer will be just as fair with his stock as he is with his hired man. He will acknowledge the fact that his cows, horses, and sheep are his capital. He cannot deal carelessly with it and expect to succeed any more than the merchant can squander his surplus of dollars and cents and expect to win. His barns will be warm, comfortable, and stored with all that will enable his stock to do their best for him."

The cause of good roads in Pennsylvania is fortunate in having a State Road Commission composed of such men as A. J. Cassatt, W. L. Elkins, Hibberd B. Worrell, James A. Beaver, H. M. Brackenridge, and H. C. Snaverley. The high character of this commission in the business world, as well as the wide experience of its members in road construction, is a guarantee that any recommendation which they present has been carefully considered, and is only given to the public after full investigation of the entire subject of road building as adapted to Pennsylvania conditions. The commission has presented its report to the Senate and House, accompanied by three bills which they recommend. The majority report recommends that the law known as the Hamilton Road Law, passed in 1897, be put into effect by repeating the twenty-first section, which provides "that a million of dollars shall be appropriated before the law becomes operative." In their report they refer to this point by saying: "This act is now inoperative, and while not so radical in many respects as may be necessary in the near future, contains provisions which are a great improvement over the existing system, and fully meets the requirements of the present time; that a large percentage of the replies to questions sent out to agricultural organizations throughout the State are favorable to the conditions of this act." The commission also prepared a bill providing for the distribution of State funds in accordance with the provisions of the aforesaid act of 1897. In their report they say: "As said act does not, however, provide sufficiently in detail for the distribution of State funds, we have also prepared with great care another bill entitled 'An act providing for the election of road supervisors and for the distribution of road appropriations for road purposes.'" The Road Commission leaves the control of the roads in the hands of the localities in every respect except so far as State appropriations are concerned, and these are to be expended by the local boards, according to plans and specifications which the State prescribes. The Speedway for Philadelphia, which has been so ardently desired by her enthusiastic road drivers, is about a certainty, the bill for that purpose having passed the House and Senate and been signed by the Governor within the past few days. Gradually the elements of slowness are disappearing from our staid old town, and by the acquisition of the Speedway another rapid stride forward has been made. We shall now behold some of our prominent veterinarians giving practical demonstrations of the knack required in speeding the 2.10 trotter and rivalling their brethren of New York and Brooklyn in the gentle art of jockeying. Already we understand two of them have prepared to enter the lists by the purchase of blue-blooded nags with the requisite amount of "go." What more convincing proof is needed that opulence comes from the practice of our profession? Thus the horse industry is stimulated in Philadelphia, and the heart of the veterinarian and the horse dealer are made glad.

Horse dealers find no difficulty in disposing of first-class horses at remunerative prices; and recent sales, such as the Belmont and Daly dispersal sales, and the C. W. Williams string at the Fasig Tipton sale, have reached high-water mark. The recent horse-shows at Philadelphia and New York were notably successful, the latter showing a financial profit of $100,000. Equally successful were the dog shows held in these two cities, and the poultry, pigeon, and cat show held in Philadelphia last fall under the auspices of the "Keystone Association," was a credit to its organizers and a means of education to many who attended. The Army Reorganization bill has been passed, and the veterinarian has been left with his original status. The Committee of Veterinarians, which had prepared the ground so thoroughly, in ploughing the last furrow, struck a ROOT, which shattered their ploughshare and necessitated a laying by for repairs. This does not mean an abandonment of the cause, however. A new ploughshare will have to be forged, perhaps shaped somewhat differently, and not cutting quite so deeply, and then let us hope that success will be attained.

In the report of the Secretary of Agriculture for the year 1900 he states that the number of abattoirs and packing-houses receiving benefit of inspection was 148 in forty-five localities as against 138 in forty-one localities the preceding year. The total post-mortem inspections were 34,737,613, and the total carcasses condemned 61,906. In the microscopical inspection of pork 999,554 carcasses were examined. Of these but 19,488, or 1.95 per cent., were found to contain living trichinæ. During the quarantine season of 1899 over a million cattle were moved under the supervision of the bureau from the district infected with the Southern cattle-tick. In Texas alone over 357,000 cattle were inspected for shipment to other sections. With regard to rabies, the Secretary declares that this disease is unfortunately on the increase in the United States, and that local authorities have in most cases not efficiently controlled its outbreaks. The article on "Rabies," by Dr. Salmon, in recent numbers of the JOURNAL, we regard as the clearest and most convincing on this subject that we have ever seen, and no thoughtful mind could have read it without being convinced that rabies has an actual and potential existence. The subject of tuberculosis is receiving the best thought of the scientist and the physician, and more light is being shed upon various phases of the question. The relative virulence of the germs of human and bovine tuberculosis on the different domestic animals has been very carefully studied by Dr. Dinwiddie, of the Arkansas Agricultural Experiment Station, and is the subject of two bulletins issued from that station. Moeller, an Austrian investigator, following the lead of Rabinovitch, a Russian scientist, thought he had discovered a vegetable origin for the bacillus tuberculosis in the so-called timothy bacillus, which is a pseudobacillus having many points of resemblance to the Koch bacillus; but experiments so far have not proved it to be virulent or to be capable of acquiring virulent properties. The Tuberculosis Convention, which met recently at Ottawa, Ontario, proposed radical legislation to limit the ravages of consumption. Resolutions were adopted in favor of imposing on national, provincial, and municipal governments the duty of applying certain methods of restricting the disease, such as the notification of health officers of every case of consumption; the prohibition of expectoration in public and private buildings; the inspection of all work-places with a view to improving the ventilation, lighting, and general sanitation thereof; the inspection of milk and meat; the establishment of homes for the care of tuberculous patients; the employment of stringent quarantine regulations against consumptive immigrants, etc. Surprise was expressed that the United States Government had refused to recognize consumption as a quarantine disease. The fact that consumption is a house ailment, and is favored by inadequate ventilation, unwholesome food, insufficient clothing, and the like, has caused it to be peculiarly afflictive in the case of the poorer people of a community.

That there are no hospitals in most cities into which consumptives are received leaves the poorer victim with little hope of care, even though the disease may be curable. In Chicago, for example, there is no general hospital, and no sort of home maintained by voluntary societies into which a tuberculous patient may go. In this city a society has for a number of years cared for a limited number of poor consumptives, but the doors of virtually all the hospitals have been closed to them. In Illinois steps have been taken to enlist the power and money of the State in the fight against the scourge. The Society for Preventing the Spread of Tuberculosis has caused to be prepared a bill which the Legislature may be prevailed upon to adopt, as the measure has the approval of the State Board of Health and of all the medical associations. It provides for an appropriation of $200,000 for the establishment of a sanitarium for the scientific treatment of consumption exclusively. It is expected that the authority of the public health officials over tuberculous patients in tenements will be increased. We hope that the time may soon come when every State shall have one or more such sanitariums.

